# Combination interventions to control the tuberculosis epidemic in the Karamoja subregion of Uganda: A modelling analysis

**DOI:** 10.1371/journal.pgph.0004853

**Published:** 2026-02-06

**Authors:** Stella Zawedde-Muyanja, Letisha Najjemba, Tadeo Nsubuga, Brenda Picho, Etwom Alfred, David Dowdy, Theresa Ryckman

**Affiliations:** 1 Department of Research, Infectious Diseases Institute, College of Health Sciences, Makerere University, Kampala, Uganda; 2 United States Agency for International Development Program for Accelerated Control of TB in Karamoja, Moroto, Uganda; 3 Division of Infectious Diseases, Department of Medicine, Johns Hopkins University School of Medicine, Baltimore, Maryland, United States of America; Burnet Institute, AUSTRALIA

## Abstract

The Karamoja subregion in northeastern Uganda is disproportionately affected by tuberculosis (TB), with an estimated TB prevalence >3 times the national average. Recently introduced health systems strengthening (HSS) interventions, including active symptom screening campaigns covering approximately 20% of the population annually, have substantially increased the number of people initiated on TB treatment, improved TB treatment completion and increased uptake of TB preventive therapy. However, to achieve further reductions in TB incidence and mortality, additional interventions are needed. We aimed to assess the impact of four additional interventions, layered onto existing HSS interventions, on TB incidence and mortality. We developed a dynamic compartmental model calibrated to TB epidemiology in Karamoja (population 1.4 million, 50% children <15 years old, 34% undernourished; estimated annual TB incidence 670 per 100,000). In addition to the existing HSS interventions, we modelled four interventions: adding chest X-ray with computer aided detection to current case-finding campaigns; increased home-based contact investigation to cover all persons diagnosed with TB plus nutritional support for undernourished persons with TB; community-wide testing and treatment of TB infection; and nutritional support to undernourished persons with TB infection. We estimated the number of TB disease episodes and deaths averted over 20 years. Over 20 years, continued implementation of HSS would reduce TB incidence by 2.4% per year to 339/100,000 (95% UI 222–475) and TB mortality by 3.2% per year to 13/100,000 (95% UI 6–20), compared to current incidence and mortality. Implementation of all four additional interventions could accelerate impact, bringing TB incidence to 161/100,000 (95% UI 61–299) and TB mortality to 5/100,000 (95% UI 2–10), thereby averting 47,450 (95% UI 18,030–76,640) TB episodes and 2,880 (95% UI 1,040–3980) TB deaths compared to HSS alone. Combination interventions to reduce undernutrition and find people with TB disease and infection can have a meaningful epidemiological impact in Karamoja.

## Introduction

The Karamoja subregion in northeastern Uganda is disproportionately affected by TB [1]. The area is mostly rural with a semi-nomadic population. Migration, which is largely limited to boys and young men, follows seasonal cyclic patterns with out-migration during the dry season and in-migration during the rainy season, when people return to farm the land. The majority of the population live in overcrowded and poorly ventilated settlements, and have limited access to healthcare services [2]. At least one-third of the population is undernourished [3]. This combination of epidemiological and demographic challenges increases transmission rates and may cause delays in case detection resulting in very high TB incidence and prevalence. A recent study estimated that the TB prevalence in this region is approximately 853/100,000 population [1], which is more than three times the national average [4].

Over the past five years, health systems strengthening (HSS) interventions have been implemented in the Karamoja region to improve TB care delivery. The current HSS interventions include healthcare worker training and supervision, deployment of rapid molecular diagnostic tests, enhanced specimen transport, and differentiated care models to enhance TB treatment initiation and completion – as well as TB screening and provision of TB preventive therapy (TPT) for contacts of patients with bacteriologically confirmed TB [5]. From 2022-2023, periodic community-based active TB case-finding using the World Health Organization (WHO) four symptom screen was layered onto these interventions as part of the nationwide Community Awareness, Screening, Testing and prevention (CAST) campaign [6]. These interventions have increased TB case notifications from 298/100,000 in 2020–445/100,000 in 2023, improved treatment completion from 53% to 84% and expanded TPT uptake for household contacts younger than five years from 23% to 89% [7].

Despite the success of these strategies, TB incidence and prevalence remain very high and additional interventions will be needed to further improve TB case-finding and increase uptake of TB preventive therapy. Further, additional interventions are needed to address socio-economic risk factors such as high rates of undernutrition that increase the likelihood of developing TB disease [8–10]. In this work, we aimed to use a dynamic mathematical model to estimate the epidemiological impact of four additional interventions on the TB burden in this region.

## Methods

### Model structure

**Fig 1:**
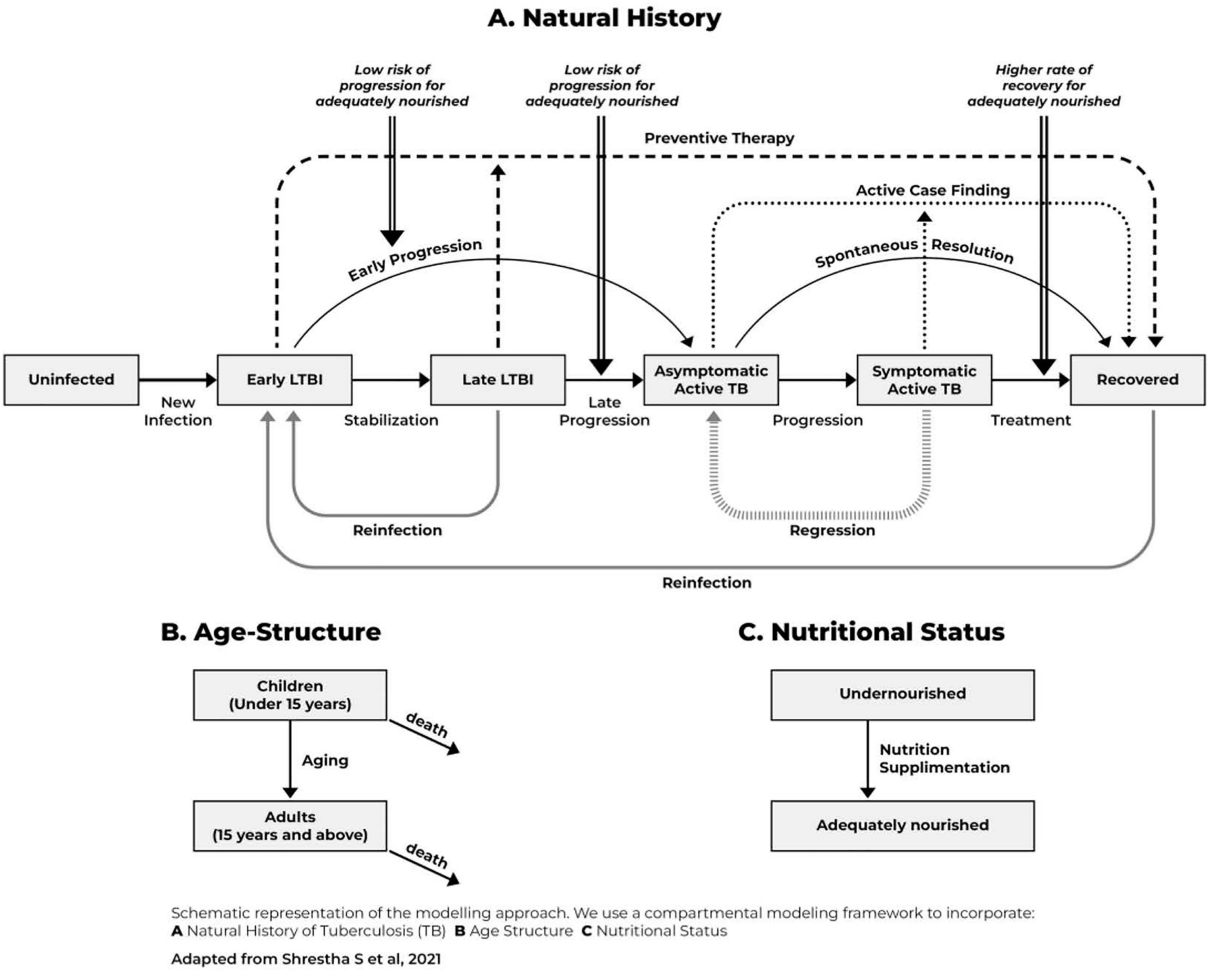
Panel A shows how the modelled population transitions between six TB-related states, with arrows representing transitions and boxes representing states. Horizontal lines show the natural progression of TB disease. The population is assumed to be uninfected at birth. Upon infection, we model transitions to the early latent TB infection (LTBI) state, where individuals spend on average two years at elevated risk of progression to TB disease before transitioning to the late latent TB infection state, where progression risks are assumed to be lower. Once individuals progress to TB disease, they start in an asymptomatic phase, from which they can spontaneously resolve to a recovered state or transition to a symptomatic state. From the symptomatic state, individuals can regress back to the asymptomatic TB state (repeated vertical bar line), be detected, treated, and cured (transition to the recovered state), or die of TB related causes. Bold grey lines show re-infection of persons with TB after cure or during late latent phase; bold dotted lines show the effect of TB preventive treatment (transitions from TB infection to recovered); and small dotted lines show the effect of active TB case finding interventions (transitions from TB disease to recovered). Double lines show how nutritional status is modelled as increasing the risk of progression and decreasing the probability of cure from the symptomatic TB state. **Panel B:** The population was subdivided into two groups based on age: children <15 years and adults ≥15 years. **Panel C:** The population was subdivided into undernourished and adequately nourished groups. People were modelled as moving from undernourished to the adequately nourished group following successful nutritional supplementation.

We adapted a deterministic compartmental model of TB in Karamoja from previous work that modelled TB interventions in India [11]. We modelled transitions between six TB-related states: an uninfected state, two states of TB infection (early and late infection), two states of active TB disease (asymptomatic and symptomatic), and a recovered state (Fig 1).

We further subdivided the population by age and nutritional status. Specially, we modelled two age strata (<15 years and ≥15 years) and two nutritional strata (undernourished and adequately nourished) with different rates of TB progression, mortality, and treatment success.

In this model, we assumed that progression to active TB is more rapid within the first two years of TB infection. Furthermore, we assumed that active TB is asymptomatic initially, progresses to symptomatic disease or resolves spontaneously, and that symptomatic TB is more infectious than asymptomatic TB. Symptomatic TB may result in death, cure through treatment, or regression to asymptomatic disease. Individuals who have TB infection or have recovered from previous TB disease can be re-infected (transition to early infection) but are assumed to have partial immunity from previous exposure.

We assumed that persons were born into the uninfected state and could exit each state due to death unrelated to TB. We further assumed that the risk for TB transmission in this population is homogenous and that the population is closed with no immigration or emigration. We did not model HIV status, since HIV prevalence is only 2.1% (95% Confidence Interval [CI] 1.3-2.8) among adults ≥15 years in Karamoja [12]. Similarly, we did not consider drug resistance because the prevalence of multidrug-resistant TB in Karamoja is very low (only 41 notified patients in 2022) [7].

We modelled initiation and completion of TPT as a transition from the TB infected states to the recovered state. We modelled active TB case-finding, successful linkage to treatment and successful treatment completion as a transition from the active TB states to the recovered state. The model simulates homogeneous population-level transmission; we modelled interventions targeting household contacts by estimating the proportion of people with TB disease and infection that were household contacts of people with diagnosed TB. This estimate was based on evidence on the prevalence of TB disease, relative prevalence of infection among household contacts and the average household size in Karamoja [13,14]. We modelled the effect of nutritional support during TB treatment as resulting in a higher rate of transition from symptomatic TB disease to the recovered state. We modelled nutritional support to persons found to be undernourished during active TB case-finding as a one-time reduction in prevalence of undernutrition.

### Model calibration

We calibrated the model to key demographic and epidemiological features of TB in the Karamoja subregion (Table 1). The region has a population of 1.4 million, 50% percent of whom are under 15 years and 34% of whom are undernourished. The region is estimated to have a TB incidence of 670/100,000 and a prevalence of 853/100,000 [1]. The annual TB related mortality is estimated to be 18.4/100,000 [15]. The case notification rate at the end of 2022 was 492/100,000, corresponding to an estimated notification-to-prevalence ratio of 57.6%.

**Table 1:** Model calibration targets.

Target	Estimate	Data Source
TB prevalence per 100,000	853 (95% CI, 689–1068)	Henry et al [1]
Annual TB incidence per 100,000	670 (95% CI, 559–810)	Henry et al [1]
Annual TB-related mortality per 100,000^β^	18.39/100,000 (95% CI, 5–35)	WHO Uganda country profiles 2012–2022 [15]
Annual TB notifications per 100,000, 2017	187 (95% CI, 166–208)	Uganda NTLP DHIS II Database [16]
Annual TB notifications per 100,000, 2022	492 (95% CI, 436–548)	Uganda NTLP DHIS II Database [16]
Proportion of prevalent TB that is asymptomatic	50% (95% CI, 42.0% - 57.9%)	Uganda National Tuberculosis Prevalence Survey [4]
Proportion of notified TB occurring among children 0–14 years^ƴ^	27% (95% CI, 22%-32%)	USAID Program for Accelerated Control of TB in Karamoja, Annual Reports 2020–2023.
Relative incidence of TB among persons with undernutrition	HR 2.23 (95% CI, 1.83 - 2.72)	Franco JV et al [17]

βAverage annual TB deaths among HIV negative persons. ^ƴ^Average annual TB notifications among children 2020–2023.

We calibrated the model using Bayesian Sampling Importance Resampling [18]. We first used Latin Hypercube Sampling to sample one million sets of model parameter values describing TB natural history, transmission, nutrition-related risks, and the current health systems strengthening interventions (S1 Table). For each parameter set, we brought the model to equilibrium over a 500-year “burn-in” phase. At the end of this phase, we modelled gradual improvements in the treatment initiation rate consistent with recent TB initiatives in the region over five years (representing 2017–2022). Modelled outputs during these five years were compared with calibration targets using normal likelihood functions parameterized to fit the target means and 95% confidence intervals. The posterior parameter distribution was estimated by sampling 10,000 posterior parameter sets from all one million prior parameter set samples with replacement, using likelihood-based sampling weights. These posterior parameter sets were then used to simulate the impact of interventions beyond 2022.

### Model interventions

Our model conceptualizes a scenario where the current HSS interventions in the Karamoja subregion are maintained but additional interventions are layered on to accelerate the decline in TB incidence. Intervention-related parameters were taken from the published literature and from programmatic data in Karamoja, and are described in more detail in S2 Table.

We conceptualized four additional interventions layered onto the HSS interventions above. These interventions were selected based on their documented ability to reduce population-level TB incidence in other settings [19–21]; capacity to augment already existing interventions; and feasibility and scalability (following consultation with the Uganda National TB and Leprosy Program/NTLP). The first intervention involved augmenting existing semi-annual active TB case-finding interventions (which cover 20% of the population each year) to detect people with asymptomatic TB, using chest X-ray (CXR) with computer-aided detection (CAD). We assumed that screening with CXR would have a sensitivity of 89% for active TB [4], that 70% of patients identified with presumptive TB would access Xpert Ultra testing, that 90% of those diagnosed with TB would start TB treatment [7].

The second intervention entailed increasing the coverage of home-based contact investigation to include all contacts of patients diagnosed with TB (regardless of the presence or absence of bacteriological confirmation) and nutritional support for TB patients with undernutrition. We assumed that 84% of contacts with presumptive TB would access Xpert Ultra testing, 3% of those tested with Xpert Ultra would be diagnosed with TB, and 90% would start TB treatment. We also assumed that 47% of household contacts would have TB infection and, based on programmatic data, 84% would be started on TPT [7] and 93% would complete treatment (with 65% efficacy [22]). We assumed that nutritional support would reduce mortality among TB patients with undernutrition by 60% [23].

Our third intervention entailed the addition of screening for TB infection (using tuberculin skin test/TST) and undernutrition among persons who screen negative on CXR during community-wide case-finding, followed by provision of TPT to TST positive persons with undernutrition. We assumed that 70% of participants would have their TST read. We made the same assumptions for TPT initiation and completion as in intervention 2 above.

Our final intervention involved providing one-time nutritional support to all undernourished persons found during the next community-wide active TB case-finding (regardless of whether they received TPT or not). We assumed a 28% reduction in the prevalence of undernutrition among those that receive nutritional support [23].

### Model outcomes

The primary outcome for this model was the total number of TB episodes averted over twenty years. The secondary outcome was the number of TB deaths averted over the same period. Outcomes under each of the 10,000 posterior parameter sets were estimated by subtracting the number of projected TB episodes and TB deaths in the intervention scenarios from the projected numbers in the comparator scenario (where only the current health systems strengthening interventions continue). We also describe annualized reductions in incidence and mortality, relative to current rates (“baseline”). Results are reported as mean values across the 10,000 posterior parameter sets, with uncertainty intervals characterized by the 2.5^th^ and 97.5^th^ percentiles across those 10,000 parameters sets.

### Sensitivity analyses

We evaluated the sensitivity of each epidemiological outcome to the value of each model parameter by (a) comparing the subsets of model simulations that contained the lowest and highest deciles of each parameter and (b) evaluating partial rank correlation coefficients between model parameters and outcomes. We also evaluated the sensitivity of the same epidemiological outcomes to the coverage of community-wide screening (10% and 50%, compared to 20% in the base case). Finally, we considered additional sensitivity analyses that reduced the durability of nutritional support (with half of those whose nutritional status was improved by intervention returning to undernourishment after one year) and proxied in- and out-migration (by replacing 5% of the population each year with a population whose TB state distribution matched the Karamoja population pre-intervention).

## Results

**Fig 2:**
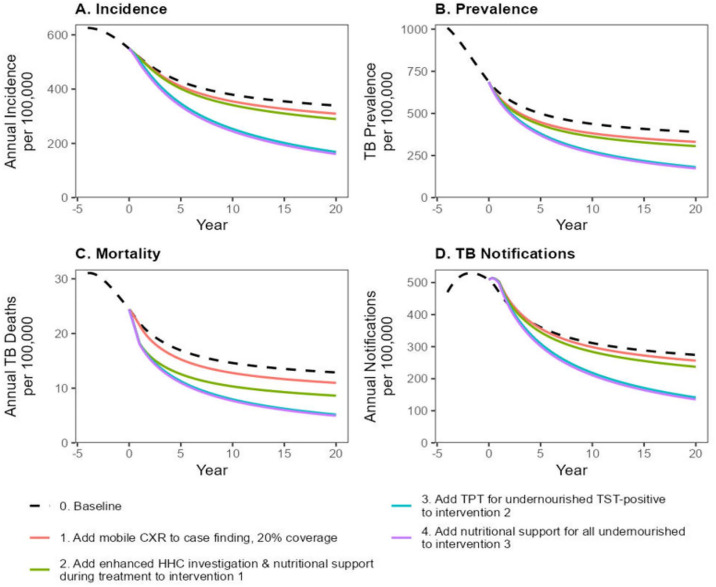
Projected declines in mean annual TB incidence (A), prevalence (B), mortality (C) and notifications (D) per 100,000 population following the layered implementation of various interventions. Black dashed lines: current health systems strengthening interventions continued over 20 years. (1) Red lines: Bi-annual community-based active TB case-finding using chest X-ray (CXR) to screen for TB achieving 20% population coverage. (2) Green lines: Adding to intervention 1 enhanced household contact (HHC) investigation and nutritional support for the undernourished during TB treatment (3) Blue: Adding to intervention 2 provision of tuberculosis preventive treatment (TPT) to all undernourished tuberculin skin test (TST) positive persons found during community-based active TB case-finding. (4) Purple: Adding to intervention 3 provision of nutritional support to all undernourished patients found during community-based active TB case finding.

The calibrated model yielded good fits to empirical data on TB epidemiology in Karamoja (S1, S2 Figs). The model estimated that the mean annual incidence of TB per 100,000 population prior to the current health systems strengthening interventions (i.e., in 2017) was 626 (95% uncertainty interval [UI] 533– 740) (Fig 2, S3 Fig). The estimated mean annual TB-related mortality rate per 100,000 was 31 (95% UI 18– 48).

During the five years of the health systems strengthening interventions, TB incidence declined at 3.7% (95% UI 1.4%– 6.0%) per year to 428 (95% UI 329–541) while TB mortality declined at 7.6% (95% UI 2.7%-15.7%) per year to 17(95% UI 9–25) (Fig 2). With the continued implementation of the current health systems strengthening interventions, our model projected TB incidence would fall by 2.4% (95% UI 1.2-3.8%) per year to 339 (95% UI 222–475) per 100,000 at 20 years while TB mortality would fall by 3.2% (95% UI 1.7-5.0%) per year to 13 (95% UI 6–20) per 100,000 at 20 years.

**Fig 3:**
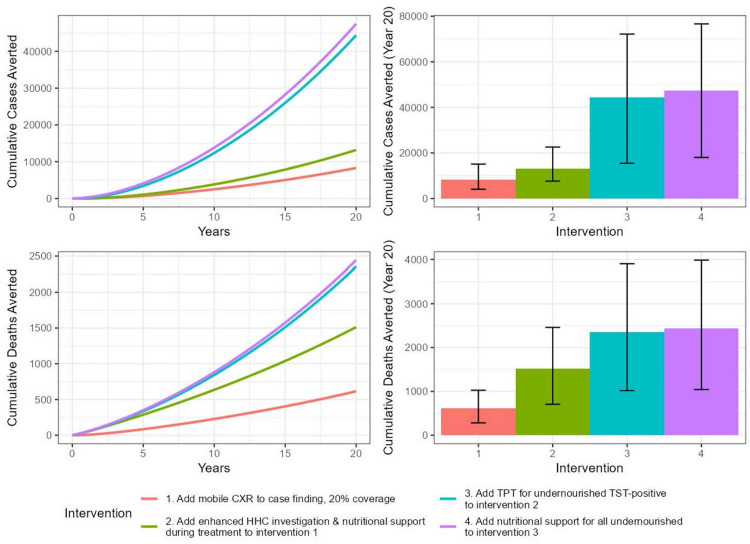
Cumulative number of TB cases and deaths averted over a 20-year period for different interventions at 20% coverage. The left panels show the time series of mean cumulative cases (top) and deaths (bottom) averted, with the x-axis representing time (starting from year 0). The right panels display stacked bar charts summarizing the total mean cases and deaths averted by year 20, with each bar segment representing the additional impact of successive interventions.

We projected that augmenting community-based TB screening with CXR + CAD (Intervention 1) would decrease TB incidence to 309 (95% UI 204–436) per 100,000 at 20 years, a 2.9% [95% UI 1.6-4.3%] annual reduction from baseline, averting 8290 (95% UI 4090–15110) TB episodes compared to current HSS interventions alone (Fig 3). This intervention would also reduce TB mortality to 11 (95% UI 5–17) per 100,000 at 20 years, a 3.9% [95% UI 2.3-5.8%] annual reduction from baseline, averting 620 (95% UI 280–1030) TB deaths over 20 years.

Layering on expanded household contact tracing and nutritional support for undernourished patients on TB treatment (Intervention 2) would further accelerate the decline in TB incidence and mortality. Over 20 years, TB incidence would reduce to 290 (95% UI 186–408) per 100,000, averting 13,200 (95% UI, 7,650–22,590) TB episodes. TB mortality would decrease to 8.6/100,000 (95% UI 4.3-14.4/100,000), averting 1,510 (95% UI 710–2,460) TB deaths. Adding provision of TB preventive therapy for undernourished persons with a positive TST during community case-finding (Intervention 3) would sharply accelerate the declines in TB incidence and mortality, reducing TB incidence to 168 (95% UI 63–317) per 100,000 and mortality to 5.2 (1.7-10.0) per 100,000 after 20 years. This combination of interventions would therefore avert 44,360 (95% UI, 15,500–72,160) TB episodes and 2,360 (95% UI, 1,020–3,900) TB deaths over 20 years. Finally, adding to Intervention 3 provision of nutritional support to all undernourished persons found during active case-finding (Intervention 4) would decrease TB incidence further to 161 (95% UI 61–299) per 100,000 and annual TB mortality to 4.9 (95% UI 1.7-9.6) per 100,000 over 20 years. The combination of all four interventions would avert an estimated 47,450 (95% UI 18,030–76,640) TB episodes and 2,880 (95% UI 1,040–3,980) TB deaths over 20 years (S4 Fig).

### Sensitivity analyses

Results were sensitive to intervention coverage. At 50% population coverage for active TB case-finding, similar interventions would produce even greater declines in incidence and mortality: the number of TB episodes and deaths averted from the combined interventions would increase to 68,160 (95% UI 35,370–97,310) and 3,300 (95% UI 1,540–5,060) over 20 years (S5 Fig). At 10% coverage, impact was reduced, with 27,860 (95% UI 10,800–48,140) episodes averted and 1780 (95% UI 520–2970) deaths averted from the combined interventions (S6 Fig).

The results were not sensitive to assumptions about the durability of nutritional support over time (S7 Fig). Greater impact on TB burden could be achieved if nutritional support was offered during each case-finding campaign (rather than only once), regardless of durability (S7 Fig).

Karamoja is home to several traditionally semi-nomadic communities and frequent migration could affect intervention impact. Given a lack of data on cross-border travel, we considered an analysis aimed at representing the upper bound on the extent of migration in the region, in which 5% of the population is replaced annually by a population with pre-intervention TB burden. This analysis suggests that, after considering migration, the impact of interventions on the TB burden in Karamoja would be less, with 37,800 (95% UI 14,780–62,970) episodes and 2330 (95% UI 980–3760) deaths averted from all interventions combined at 20 years (S8 Fig).

**Fig 4:**
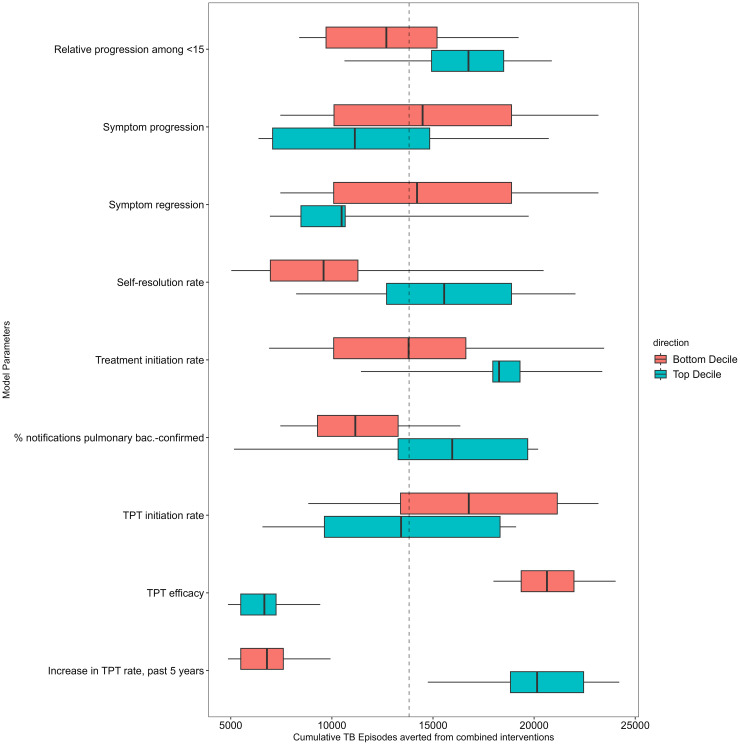
Cumulative TB cases averted across variation in selected model parameters. The boxplots display the distribution of cases averted across parameter deciles (when outcomes are estimated using only the subset of posterior parameters for which a given parameter value is within the bottom and top decile of its full posterior distribution), with the dashed line indicating the mean cumulative cases averted across all posterior model parameters. Parameters are ranked by their influence, with higher variability reflecting that variation in this parameter has a greater impact on estimated cases averted. Only the nine most influential parameters are shown.

Probabilistic sensitivity analysis revealed that the most influential model parameter for the primary outcome (cumulative TB episodes averted from the combined interventions) was the efficacy of TPT. Other influential parameters included the proportion of eligible patients started on TPT, the extent to which TPT has already been scaled up in Karamoja under current activities, the proportion of all TB patients notified that are pulmonary bacteriologically confirmed, the TB treatment initiation rate among patients diagnosed with TB, and the proportion of patients with presumptive TB who receive diagnostic testing during case-finding (Fig 4, S9, S10 Figs) Parameters that were highly correlated with the secondary outcome (cumulative deaths averted) included: the proportion of patients with presumptive TB who receive diagnostic testing, TB treatment initiation rates among those diagnosed with TB, the increased odds of death during treatment among people with undernutrition, and TB case fatality ratios (S9, S10 Figs).

## Discussion

Our modelling analysis shows that the current health systems strengthening interventions in the Karamoja subregion have resulted in a notable decline in TB incidence and mortality over the past five years, and will continue to do so over the next 20 years. Our model estimates that additional interventions that include active TB case-finding using CXR, expansion of TPT, and nutrition supplementation will further reduce TB incidence by 39% over five years. Over a twenty-year period, these interventions will reduce incidence by 71%, averting 47,500 TB episodes and 2400 deaths over twenty years and bringing the Karamoja subregion closer to controlling the TB epidemic.

Current CAST-TB case-finding campaigns typically cover 20% of households and involve symptom-based TB screening, testing of symptomatic patients and treatment of those diagnosed with TB. The results of our modelling study show that augmenting these campaigns with CXR-based screening, irrespective of symptoms, results in an additional reduction in TB burden. This aligns with the current NTLP strategic objective to expand the use of chest X-ray–based screening to improve TB case finding [24]. Our estimate of the impact on incidence of symptom-agnostic case-finding with (Intervention 3) or without TPT (Intervention 1) is greater than that of Shrestha et al. [11], who modelled a one-time intervention rather than repeated rounds of screening, and less than that of Dye et al. [21], who assumed 100% intervention coverage. Results are comparable to Ragonnet et al.’s [25] estimates of a roughly 50% reduction in incidence after 20 years from annual screening in the Marshall Islands, although our estimates of the impact of case-finding with TPT are lower, possibly reflecting lower population coverage in our model. In addition to generating Karamoja-specific evidence, our study adds to previous literature by also illustrating the impact of incorporating nutritional support into a package of TB-specific interventions.

In this analysis, the expanded provision of TPT, particularly as part of community screening, markedly increased the impact of community-based case finding. Community-wide provision of TPT targeted to those with TST positivity and undernutrition, while likely to be resource-intensive, greatly increased the number of TB episodes (44,000 vs 13,200) and deaths (2300 vs 1510) averted during this period. TPT is already provided to household contacts of pulmonary bacteriologically-confirmed patients in Karamoja; expansion to all household contacts (as part of Intervention 2) had relatively less impact than population-wide expansion.

However, population-level TPT scaleup remains challenging. There are barriers to scale up at the structural level (e.g., healthcare worker capacity, limited availability of LTBI testing) [26,27] and at the patient-level barriers (e.g., fear of potential side-effects, long waiting times at clinics, and costs to travel to health facilities for testing and medicine refills) [28,29]. Recently, TB-specific skin tests (TBSTs), whose sensitivity and specificity are comparable to Interferon Gamma Release Assays (IGRAs), have become available for widespread use [30]. These tests are particularly suited for high burden settings because they do not cross-react with BCG, cost considerably less than IGRA and do not need laboratory infrastructure [31]. They can therefore be placed and interpreted in community settings, making it possible to implement targeted TB preventive therapy interventions on a larger scale. Finally, the use of shorter TPT regimens (e.g., 3HP) combined with home-based delivery can also contribute to improved uptake and completion [32–34].

Undernutrition is the leading global population-level risk factor for TB and is estimated to more than double the risk of incident TB and increase the risk of unfavourable outcomes while on TB treatment by 50% [17,35]. In 2023, 8% of all active TB in sub-Saharan Africa and in Uganda was attributed to undernutrition [36]. Nutritional support should therefore be considered a critical intervention for TB control in settings like Karamoja where undernutrition is prevalent. Recent evidence from India has shown that nutritional supplementation significantly reduces the risk of progression to active TB among household contacts of patients diagnosed with TB and is cost effective (229 USD per disability-adjusted life year averted) [37]. Additional research on socially acceptable and sustainable means of providing nutritional support for this region should therefore be undertaken. This may involve working with other programs outside the health sector.

Our findings were dependent on all four interventions being delivered simultaneously and were sensitive to intervention coverage, as well as TPT initiation, TB treatment initiation, and the proportion of patients with presumptive TB who receive diagnostic testing. Although rapid and sustained scaleup of several new TB interventions has already been achieved in Karamoja, sustained impact of these interventions will depend on their continued delivery in Karamoja, including overcoming any logistical or financial constraints. This may become challenging, for example as vertical disease programs are replaced by integrated primary care interventions. On the other hand, as has previously been seen with the integration of TB and HIV care, integration may lead to cost efficiencies [38] which may increase or sustain coverage [39].

Our study has important strengths. First, we developed a Karamoja-specific model with parameters informed by data from this very high-burden region. Our results are expected to generalize to similar high-TB-burden, hard-to-reach, and food-insecure populations in Africa (e.g., pastoralists in the Turkana, horn of Africa and Sahel regions). Second, our inclusion of nutritional interventions highlights the importance of a multisectoral response in the fight against TB through addressing its social determinants. Finally, we targeted the provision of TPT to persons who were household contacts of patients with active TB or those who were found to be TST positive and undernourished after community-based screening. This approach makes this intervention more feasible to implement.

As with any modelling analysis, these findings also have limitations. We modelled a closed homogeneous population with no immigration or emigration. The Karamoja subregion borders Kenya and South Sudan, and some areas are inhabited by seasonal nomads who move in and out of the area in search of pasture. As our sensitivity analysis shows, substantial periodic movement in and out of the region could reduce the impact of additional interventions. However, this analysis may overestimate the extent of cross-border migration. Furthermore, out-migration may have benefits for surrounding areas that were not modelled here (i.e., reducing transmission to bordering areas to which residents of Karamoja might emigrate).

Second, we modelled nutritional support as a permanent transition to the adequately nourished compartments. Evidence from community-based treatment programs in Africa show about one in ten children treated for undernutrition eventually relapse [40,41]. However, our sensitivity analysis showed that the impact on TB episodes and deaths averted was not affected by the durability of nutritional protection. Third, as with any modelling analysis, model parameters and projections depend on the data used in calibration. While most data were Karamoja-specific, estimates of TB mortality and the impact of undernutrition on TB incidence were taken from national and international sources, respectively. Therefore, it is possible that the model has under or overestimated baseline TB mortality and the impact of nutritional interventions. Finally, like many TB modelling studies, our projections are subject to wide uncertainty intervals, largely reflecting uncertainty in underlying data on TB burden, natural history and population dynamics. Despite these inherent limitations of TB models, results from modelling studies can be useful for informing high-level policy decisions, as the approximate magnitude and relative impact of different strategies remains consistent despite uncertainty in the absolute burden of TB averted.

Our study presents some opportunities for future research. We did not include a framework for monitoring intervention effectiveness or coverage. Should these interventions be implemented, such a framework would help validate the model assumptions and allow for iterative refinement as new data becomes available. Additionally, future research to estimate the cost of implementing these interventions could demonstrate potential savings to the healthcare system (in the form of fewer people treated as incidence declines) while also providing estimates of cost-effectiveness.

## Conclusion

In conclusion, our modelling study suggests that a combination of interventions to improve active TB case finding, expand access to TPT, and provide nutritional support can reduce TB incidence in the Karamoja subregion by at least 50% and TB mortality by at least 65% over 20 years. The majority of the projected impact on incidence was achieved through increased access to TPT and realized over the first five years of implementation. In the long term, successful TB control in this region will depend heavily on achieving and sustaining high population-level coverage across a multifaceted combination of interventions.

## Supporting information

S1 Table Model prior parameter distributions and values. (DOCX)

S2 Table Intervention parameters. (DOCX)

S1 Fig Density plots showing the distribution of posterior model outputs (x-axis) compared to target values. (1) Solid vertical line represents the target mean, (2) Dashed lines indicate the lower and upper bounds of the target range. Each subplot corresponds to a different empirical target, illustrating the spread and central tendency of the simulated posterior outcomes relative to the predefined calibration targets.
(TIF)

S2 Fig Density plots illustrating the distributions of posterior model parameters (x-axis) across simulations. The dashed vertical lines represent the lower and upper bounds of the prior parameter ranges. Each subplot corresponds to a different parameter, showing how parameters were informed via the calibration process.
(TIF)

S3 Fig Projected declines in mean annual TB incidence (A), prevalence (B), mortality (C) and notifications (D) per 100,000 population following the layered implementation of various interventions with uncertainty bands. Black dashed lines: current health systems strengthening interventions continued over 20 years. (1) Red lines: Bi-annual community-based active TB case-finding using chest X-ray (CXR) to screen for TB achieving 20% population coverage. (2) Green lines: Adding to intervention 1 enhanced household contact (HHC) investigation and nutritional support for the undernourished during TB treatment (3) Blue: Adding to intervention 2 provision of tuberculosis preventive treatment (TPT) to all undernourished tuberculin skin test (TST) positive persons found during community-based active TB case-finding. (4) Purple: Adding to intervention 3 provision of nutritional support to all undernourished patients found during community-based active TB case finding.
(EPS)

S4 Fig Projected declines in mean annual TB incidence (A), prevalence (B), mortality (C), and notifications(D) per 100,000 population following the layered implementation of various interventions. Black dashed lines: current health systems strengthening interventions continued over 20 years (1) red lines: Bi-annual community- based active TB case-finding using CXRs to screen for TB achieving 50% population coverage. (2) Green lines: Adding to intervention 1 Enhanced contact investigation and nutritional support for the undernourished during TB treatment (3) Blue: Adding to intervention 2 Providing TPT to all undernourished TST positive persons found during community-based active TB case-finding and (4) Purple: Adding to intervention 3 Providing nutritional support to all undernourished patients found during community-based active TB case finding.
(EPS)

S5 Fig Cumulative number of TB episodes and deaths averted over a 20-year period for different interventions at 50% coverage. The left panels show the time series of cumulative episodes (top) and deaths (bottom) averted, with the x-axis representing time (starting from year 0). The right panels display stacked bar charts summarizing the total episodes and deaths averted by year 20, with each bar segment representing the additional impact of successive interventions.
(EPS)

S6 Fig Projected declines in mean annual TB incidence (A), prevalence (B), mortality (C), and notifications (D) per 100,000 population following the layered implementation of various interventions for different intervention coverages Black lines: current health systems strengthening interventions continued over 20 years (1) red lines: Bi-annual community- based active TB case-finding using CXRs to screen for TB. (2) Green lines: Adding to intervention 1 Enhanced contact investigation and nutritional support for the undernourished during TB treatment (3) Blue: Adding to intervention 2 Providing TPT to all undernourished TST positive persons found during community-based active TB case-finding and (4) Purple: Adding to intervention 3 Providing nutritional support to all undernourished patients found during community-based active TB case finding. PS: bold lines represent 10% coverage; dash lines represent 20% coverage; and dash-dot lines represent 50% coverage.
(EPS)

S7 Fig Projected declines in mean annual TB incidence (A), prevalence (B), mortality (C), and notifications (D) per 100,000 population following the layered implementation of various interventions for different nutrition scenarios (1) black line: baseline scenario. (2) purple line: Adding to intervention 3, one-time nutritional support for all undernourished (3) yellow: Adding to Intervention 3, bi-annual nutritional support for all undernourished. PS: bold lines: assuming permanent transition to adequately nourished; dash lines: assuming half of those benefiting from nutritional support transition back to undernourished after 1 year.
(EPS)

S8 Fig Projected declines in mean annual TB incidence (A), prevalence (B), mortality (C), and notifications (D) per 100,000 population following the layered implementation of various interventions for different intervention coverages Black lines: current health systems strengthening interventions continued over 20 years (1) red lines: Bi-annual community- based active TB case-finding using CXRs to screen for TB achieving 20% population coverage. (2) green lines: Adding to intervention 1, enhanced contact investigation and nutritional support for the undernourished during TB treatment. (3) Blue lines: Adding to intervention 1, providing TPT to all undernourished TST positive persons found during community-based active TB case-finding (4) Purple: Adding to intervention 3, providing nutritional support to all undernourished patients found during community-based active TB case finding. PS: bold lines represent a closed population; dash lines an open population assuming 5% out-migration.
(EPS)

S9 Fig Cumulative TB episodes averted across variation for all model parameters. The boxplots display the distribution of episodes averted across parameter deciles (when outcomes are estimated using only the subset of posterior parameters for which a given parameter value is within the bottom and top decile of its full posterior distribution), with the dashed line indicating the mean cumulative episodes averted across all posterior model parameters.
(EPS)

S10 Fig Heatmap displaying the Partial Rank Correlation Coefficients (PRCCs) for key model parameters influencing cumulative TB episodes averted and cumulative TB deaths averted under different intervention scenarios. Warmer (red) colors indicate a positive correlation, while cooler (blue) colors represent a negative correlation. The x-axis shows intervention labels, and the y-axis lists model parameters. Higher absolute values of PRCC indicate stronger associations between parameter variations and model outcomes.
(TIF)

## Abbreviations

ACF: Active TB case finding
CAD: Computer-aided diagnosis
CAST: Community Awareness, Screening, Testing and prevention
CI: Confidence Interval
CXR: Chest X-ray
HIV: Human Immunodeficiency Virus
HSS: Health Systems Strengthening
LTBI: Latent TB Infection
NTLP: National TB and Leprosy Program
TB: Tuberculosis
TPT: TB Preventive Therapy
TST: Tuberculin skin test
WHO: World Health Organisation
