# Supplementary material for: Combination interventions to control the tuberculosis epidemic in the Karamoja subregion of Uganda: A modelling analysis
Source: PLOS Glob Public Health. 2026 Feb 6;6(2):e0004853. doi: 10.1371/journal.pgph.0004853 (PMC12880685; doi:10.1371/journal.pgph.0004853)
Supplement: S1 Table — (DOCX) [file pgph.0004853.s001.docx]

**S1_Table: Model Prior Parameter Distributions and Values**

| **Parameter** | **Estimate** | **Distribution** | **Data Source** |
| --- | --- | --- | --- |
| Treatment outcomes: % success | 91% [95% CI 84-94%] | Multinomial Distribution, size=250, probability=90%* | Uganda NTLP DHIS II Database^17^ |
| Treatment outcomes: % dying during treatment | 3% [95% CI 2-5%] | Multinomial Distribution, size=250, probability=3.5%* | Uganda NTLP DHIS II Database^17^ |
| Treatment outcomes: % failing treatment | 6% [95% CI 2-11%] | Multinomial Distribution, size=250, probability=6.5%* | Uganda NTLP DHIS II Database^17^ |
| Relative probability of unfavorable treatment outcomes among people with undernutrition | RR 1.50 [95% CI 1.15-1.86] | Log normal, meanlog=0.40, sdlog=0.12 | Sinha P et al, Impact of undernutrition on tuberculosis treatment outcomes in India: a multicenter, prospective, cohort analysis. Clinical Infectious Diseases^35^. |
| Relative odds of death while on TB treatment among people with undernutrition | OR 1·8, (95% CI 1·1–2·7). | Log normal, meanlog=0.56, sdlog=0.22 | Zachariah R et al, Moderate to severe malnutrition in patients with tuberculosis is a risk factor associated with early death. Transactions of the Royal Society of Tropical Medicine and Hygiene^42^. |
| Proportion of the population that is under 15 years | 50% | No uncertainty modeled | Uganda Household Survey 2020 |
| Annual birth rate | 45 births/1000 population/year | No uncertainty modeled | Uganda Demographic and Health Survey 2022.^3^ |
| Annual all-cause mortality rate,  < 15 years | 5.11 deaths/1000 population/year | No uncertainty modeled | Uganda Demographic and Health Survey 2022^3^ |
| Annual all-cause mortality rate, 15+ years | 18.0 deaths/1000 population/year | No uncertainty modeled | Uganda Demographic and Health Survey 2016^3^  WHO Life Tables |
| TB-specific mortality rate, symptomatic untreated TB | Calibrated | Uniform Distribution, mean=0.01, max=0.1 | Wide, uninformed prior (informed by calibration) |
| Symptomatic TB transmission rate | Calibrated | Uniform Distribution, mean=5, max=30 | Wide, uninformed prior (informed by calibration) |
| Relative transmissibility of asymptomatic TB | Calibrated | Uniform Distribution, mean=0.35, max=1 | Wide, uninformed prior (informed by calibration) |
| Relative risk of being re-infected if previously infected | Calibrated | Uniform Distribution, mean=0.3, max=0.5 | Wide, uninformed prior (informed by calibration) |
| Relative risk of progression with undernutrition | Calibrated | Uniform Distribution, mean=1, max=5 | Wide, uninformed prior (informed by calibration) |
| Relative risk of progression among children < 15 | Calibrated | Uniform Distribution, mean=0.5, max=1 | Wide, uninformed prior (informed by calibration) |
| Early progression rate, adequately nourished adults | Calibrated | Uniform Distribution, mean=0.03, max=0.06 | Wide, uninformed prior (informed by calibration) |
| Late reactivation rate, adequately nourished adults | Calibrated | Uniform Distribution, mean=0.001, max=0.005 | Wide, uninformed prior (informed by calibration) |
| Symptom progression rate | Calibrated | Uniform Distribution, mean=1, max=4 | Wide, uninformed prior (informed by calibration) |
| Rate of symptom regression, relative to symptom progression | Calibrated | Uniform Distribution, mean=0.5, max=1 | Wide, uninformed prior (informed by calibration) |
| Rate of self-resolution | Calibrated | Uniform Distribution, mean=0.1, max=0.75 | Wide, uninformed prior (informed by calibration) |
| Treatment initiation rate | Calibrated | Uniform Distribution, mean=0.6, max=1 | Wide, uninformed prior (informed by calibration) |
| Annual increase in the treatment initiation rate during the last 5 years of burn-in (pre-intervention period) | Calibrated | Uniform Distribution, mean=0, max=0.3 | Wide, uninformed prior (informed by calibration) |

*The treatment outcome probabilities (of success, death, and failure) were sampled together via a multinomial distribution with size=250 and probabilities equal to 90%, 3.5%, and 6.5%, respectively.
