# Supplementary material for: Combination interventions to control the tuberculosis epidemic in the Karamoja subregion of Uganda: A modelling analysis
Source: PLOS Glob Public Health. 2026 Feb 6;6(2):e0004853. doi: 10.1371/journal.pgph.0004853 (PMC12880685; doi:10.1371/journal.pgph.0004853)
Supplement: S2 Table — (DOCX) [file pgph.0004853.s002.docx]

**S2_ Table: Intervention Parameters**

| **Parameter** | **Estimate** | **Distribution** | **Data Source** |
| --- | --- | --- | --- |
| **No Intervention scenario: Background health systems strengthening** | | | |
| Increase in the treatment initiation rate from 2017-2022 | Prior: 0.15 (95% CI 0.01-0.29)  Posterior: 0.20 (95% CI 0.08-0.29) |  | Calibrated (from a wide, uninformed prior) |
| Percent of the population that is a household contact of a person with notified TB | Prior: 1.3% (95% CI 0.8-1.7%)  Posterior: 1.3% (95% CI 0.9-1.7%) | Normal distribution, mean=0.013, sd=0.002  Normal distribution, mean=0.013, sd=0.002 | Informed via calibration; prior estimated from Uganda National Household Survey 2019-2020^43^ and  Uganda NTLP DHIS II Database^17^ |
| Relative prevalence of TB infection among households with a person with TB | Prior: 3.0 (95% CI: 2.7-3.3)  Posterior: 3.0 (95% CI: 2.7-3.3) | Normal distribution, mean=1.6, sd=0.13  Normal distribution, mean=3.0, sd=0.16 | Informed via calibration; prior estimated from Whalen et al. Secondary Attack Rate of Tuberculosis in Urban Households in Kampala, Uganda^13^. |
| % total TB notifications that are bacteriologically-confirmed | Prior: 66% (95% CI, 55%-71%)  Posterior: 66% (95% CI, 57-73%) | Beta distribution, alpha=86.3, beta=44.6  Beta distribution, alpha=86.3, beta=44.6 | Informed via calibration; prior estimated from the 2023 Karamoja Annual Report^7^ |
| TPT initiation | Prior: 84% (95% CI, 83.8%-84.7%)  Posterior: 84% (95% CI, 83.5-84.4%) | Normal distribution, mean=0.84, sd=0.002  Normal distribution, mean=0.84, sd=0.002 | Informed via calibration; prior estimated from the Uganda NTLP DHIS II Database^17^ |
| TPT completion | Prior: 92.9% (95% CI, 90.2%-94.9%)  Posterior: 93.0% (95% CI, 90.9 – 95.3%) | Normal Distribution, mean=0.929, sd=0.012  Normal Distribution, mean=0.93 sd=0.012 | Informed via calibration; prior estimated from Semitala FC et al, Completion of isoniazid–rifapentine (3HP) for tuberculosis prevention among people living with HIV: Interim analysis of a hybrid type 3 effectiveness–implementation randomized trial^44^. |
| TPT Efficacy | Prior: 65% (95% CI 12% - 90%)  Posterior: 65% (95% CI 20–96%) | Beta distribution, alpha=3.08, beta=1.66  Beta distribution, alpha=2.70, beta=1.46 | Informed via calibration; prior estimated from Pease C et al, Efficacy and completion rates of rifapentine and isoniazid (3HP) compared to other treatment regimens for latent tuberculosis infection: a systematic review with network meta-analyses^22^. |
| **Intervention 1: Chest X-ray to the community-based case finding screening algorithm** | | | |
| Estimated coverage of case finding | 20% | No uncertainty modeled | Karamoja annual report CAST TB performance 2023 |
| Sensitivity of CXR | 88.8% (95% CI 83.8-93.6) | Normal distribution, mean=0.888, sd= 0.02 | Uganda National TB Prevalence Survey^4^ |
| % presumptive TB detected by CXR who access GeneXpert testing | 70% (95% CI, 69.2% -70.7%) | Beta Distribution, alpha=5656, beta=9112 | Assumption: Using mobile vans fitted with CXR plus GeneXpert testing would result in at least 70% of presumptive TB accessing testing |
| % detected by CXR who initiate treatment | 90% (95% CI 72% -100%) | Beta Distribution, alpha=16.2, beta=1.82 | Karamoja annual report CAST TB performance 2023 |
| **Intervention 2: Enhanced household contact investigation of all patients routinely diagnosed with TB (regardless of the presence or absence of bacteriological confirmation)^β^**  **plus nutritional support for undernourished people on TB treatment to reduce case fatality/increase treatment success** | | | |
| Relative prevalence of TB infection among households with a person with TB | 3.0 (95% CI: 2.7-3.3) | Calibrated | Same as no intervention scenario |
| % HH contacts with presumptive TB who access GeneXpert testing | 83% (95% CI, 80.0% -86.4%) | Beta Distribution, alpha=438.5, beta=89.8 | 2023 Karamoja Annual Report^7^ |
| Percent of household contacts of both bacteriologically-confirmed and non-bacteriologically confirmed index TB patients that have TB | 1.6% (95% CI1.4-1.9%) | Normal, mean=0.016, sd=0.0013 | Kisamba et al, Tuberculosis yield among contacts of non- bacteriologically confirmed index TB patients in the urban setting of central Uganda^14^. |
| % total TB notifications that are non-bacteriologically-confirmed | 34% (95% CI, 29.0%-55.3%) | Calibrated | Same as no intervention scenario |
| TPT initiation | 84% (95% CI, 83.5-84.4%) | Calibrated | Same as no intervention scenario |
| TPT completion | 93.0% (95% CI, 90.9 – 95.3%) | Calibrated | Same as no intervention scenario |
| TPT Efficacy | 65% (95% CI 20–96%) | Calibrated | Same as no intervention scenario |
| Relative risk of unsuccessful treatment outcomes with nutritional support (among people with undernutrition) | 67% (95% CI 56% - 92%) | Beta distribution, alpha=17.2, beta=8.4 | Bhargava A et al, Nutritional support for adult patients with microbiologically confirmed pulmonary tuberculosis: outcomes in a programmatic cohort nested within the RATIONS trial in Jharkhand, India^23^. |
| **Intervention 3: Screening for undernutrition and LTBI among persons who screen negative on CXR during community-wide active TB case-finding interventions followed by provision of TPT to TST positive persons.** | | | |
| Sensitivity of mid-upper-arm circumference (MUAC) in detection undernutrition (in both children and adults) | 92.7% (95% CI, 80.7%-98.5%) | Beta distribution, alpha=26, beta=2 | Thorup L et al, Mid-upper arm circumference as an indicator of underweight in adults: a cross-sectional study from Nepal^45^ |
| Percent of the population seen during active TB case-finding that is undernourished. | 34% | No uncertainty modeled | Equals the modeled prevalence of undernutrition, based on  Uganda National Household Survey 2019-2020^43^  Uganda NTLP DHIS II Database^17^ |
| Percent of undernourished persons who will have their TB infection test read | 70% (95% CI, 69.0% -86.4%) |  | We assumed that the proportion of patients who had their TST read would be 20% lower under programmatic conditions than that observed during a recently completed implementation research study in Uganda [Manuscript in progress] |
| TPT initiation | 84% (95% CI, 83.5-84.4%) | Calibrated | Same as no intervention scenario |
| TPT completion | 93.0% (95% CI, 90.9 – 95.3%) | Calibrated | Same as no intervention scenario |
| TPT Efficacy | 65% (95% CI 20–96%) | Calibrated | Same as no intervention scenario |
| **Intervention 4: Add nutritional support for undernourished people found during active TB case-finding regardless of TST status** | | | |
| Percent reduction in the prevalence of undernutrition, among undernourished individuals receiving nutritional supplementation | 28% (95% CI, 23% -33%) | Estimated empirically from distributions among control and intervention groups in Bhargava et al. | Bhargava A et al, Nutritional support for adult patients with microbiologically confirmed pulmonary tuberculosis: outcomes in a programmatic cohort nested within the RATIONS trial in Jharkhand, India^23^. |

**^β^**Product of estimated coverage of case finding (% population screened, as an annual rate), TPT acceptance/initiation rate, TPT efficacy, TPT completion. Note: across all intervention scenarios, we assumed the same TB treatment outcomes once an individual initiates TB treatment.
