# Supplementary figures and images for: Combination interventions to control the tuberculosis epidemic in the Karamoja subregion of Uganda: A modelling analysis
Source: PLOS Glob Public Health. 2026 Feb 6;6(2):e0004853. doi: 10.1371/journal.pgph.0004853 (PMC12880685; doi:10.1371/journal.pgph.0004853)

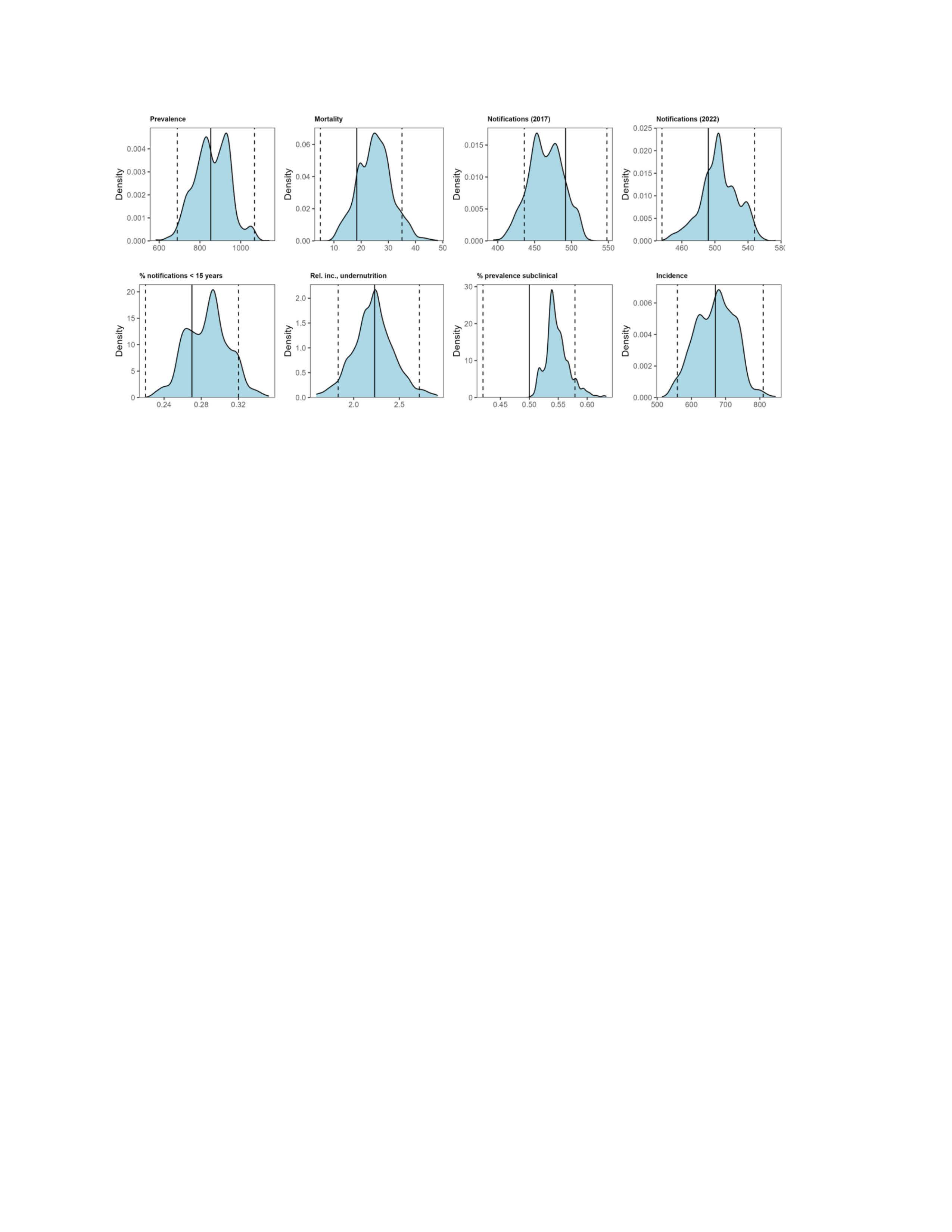

Supplement: S1 Fig — (1) Solid vertical line represents the target mean, (2) Dashed lines indicate the lower and upper bounds of the target range. Each subplot corresponds to a different empirical target, illustrating the spread and central tendency of the simulated posterior outcomes relative to the predefined calibration targets. (TIF) [file pgph.0004853.s003.tif]

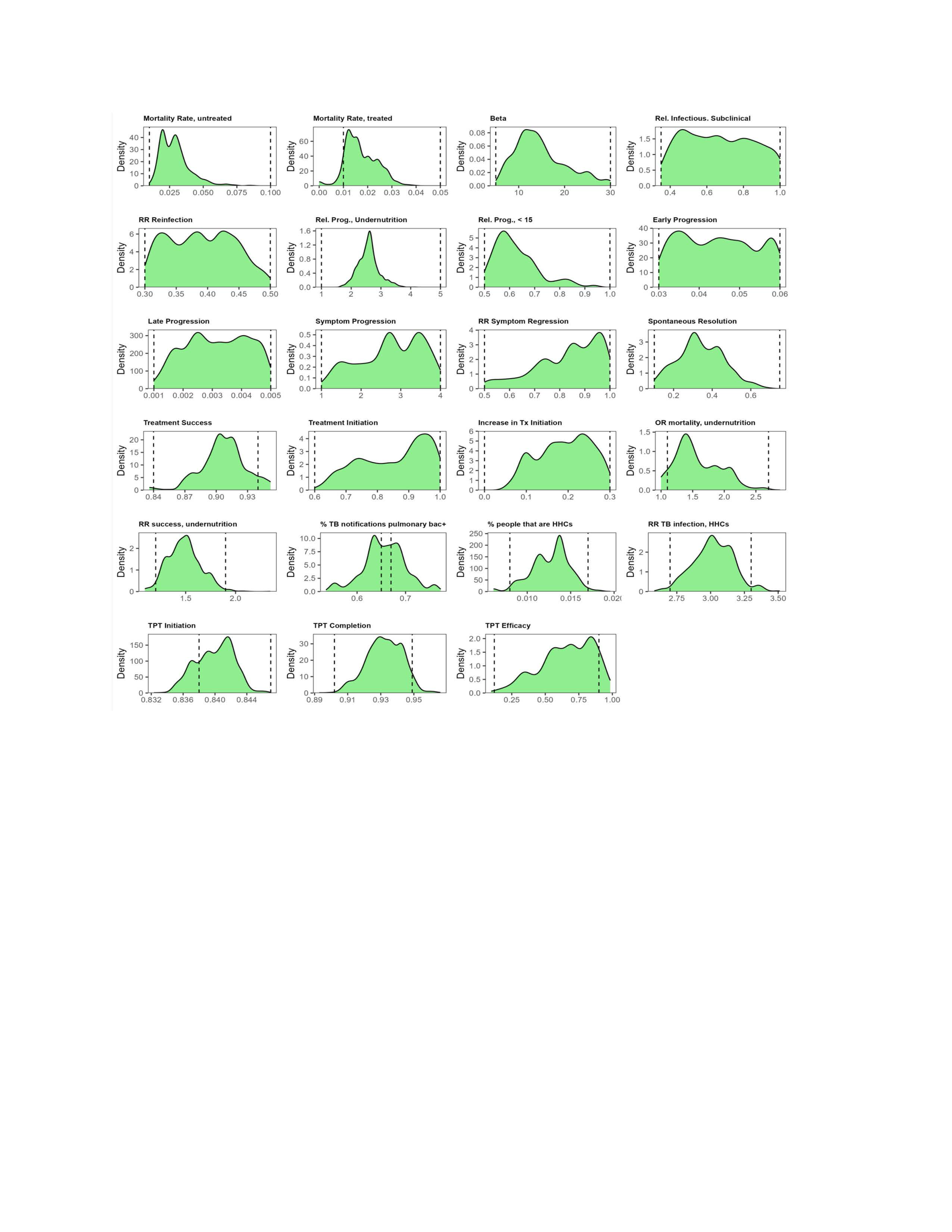

Supplement: S2 Fig — The dashed vertical lines represent the lower and upper bounds of the prior parameter ranges. Each subplot corresponds to a different parameter, showing how parameters were informed via the calibration process. (TIF) [file pgph.0004853.s004.tif]

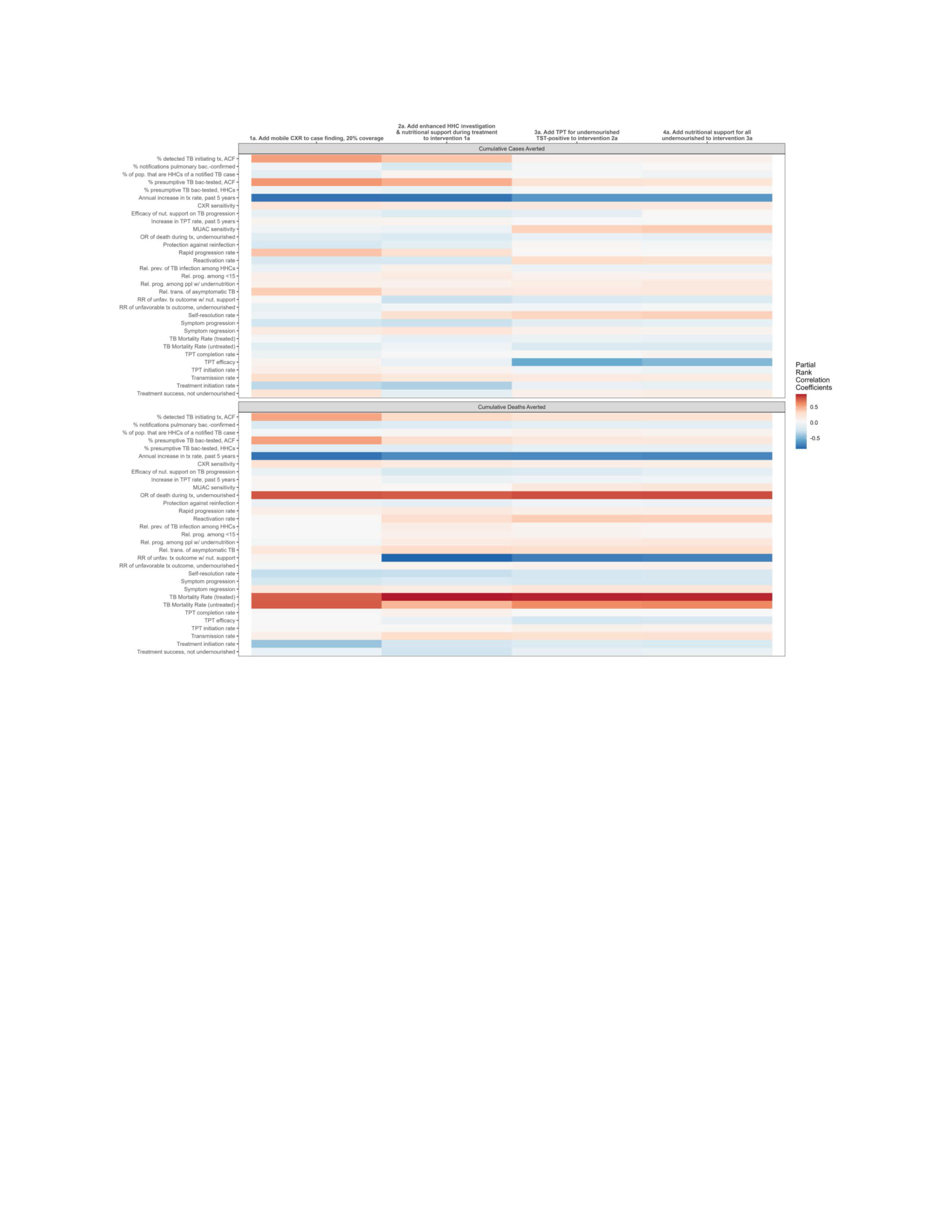

Supplement: S10 Fig — Warmer (red) colors indicate a positive correlation, while cooler (blue) colors represent a negative correlation. The x-axis shows intervention labels, and the y-axis lists model parameters. Higher absolute values of PRCC indicate stronger associations between parameter variations and model outcomes. (TIF) [file pgph.0004853.s012.tif]
